# Genetic and phenotypic diversity in 2000 years old maize (*Zea mays* L.) samples from the Tarapacá region, Atacama Desert, Chile

**DOI:** 10.1371/journal.pone.0210369

**Published:** 2019-01-30

**Authors:** Ale Vidal Elgueta, Luis Felipe Hinojosa, María Fernanda Pérez, Gioconda Peralta, Mauricio Uribe Rodríguez

**Affiliations:** 1 Departamento de Ecología, Pontificia Universidad Católica de Chile, Santiago, Chile; 2 Laboratorio de Paleoecología, Facultad de Ciencias Biológicas, Universidad de Chile, Santiago, Chile; 3 Instituto de Ecología y Biodiversidad (IEB), Santiago, Chile; 4 Facultad de Ciencias Biológicas, Pontifica Universidad Católica de Chile, Chile; 5 Departamento de Antropología, Facultad de Ciencias Sociales, Universidad de Chile, Santiago, Chile; New York State Museum, UNITED STATES

## Abstract

The evolution of maize (*Zea mays* L.) is highly controversial given the discrepancies related to the phenotypic and genetic changes suffered by the species, the incidence of human groups and the times in which these changes occurred. Also, morphological and genetic traits of crops are difficult to evaluate in the absence of fossils macro-botanical remains. In contrast in the Tarapacá region (18–21° S), Atacama Desert of Chile, prehispanic settlements (ca. 2500–400 yr BP) displayed extensive maize agriculture. The presence of archaeological macro-botanical remains of maize provided a unique opportunity to study the evolution of this crop, covering a temporal sequence of at least 2000 years. Thus, in this study, we ask how the morphological and genetic diversity of maize has varied since its introduction during prehispanic times in the Tarapacá region. To answer this, we measured and compared morphological traits of size and shape between archaeological cobs and kernels and 95 ears from landraces. To established genetic diversity eight microsatellite markers (SSR) were analyzed in archaeological and modern kernels. Genetic diversity was estimated by allelic frequency rates, the average number of alleles per locus, observed heterozygosity (Ho) and expected heterozygosity (He). Differences between populations and genetic structure were estimated by fixation index F_ST_ and STRUCTURE analysis. Our results indicate significant phenotypic differences and genetic distance between archaeological maize and landraces. This result is suggestive of an introduction of new varieties or drastic selective changes in modern times in Tarapacá. Additionally, archaeological maize shows a low genetic diversity and a progressive increase in the size of ears and kernels. These results suggest a human selection during prehispanic times and establish that prehispanic farmers played an important role in maize development. They also provide new clues for understanding the evolutionary history of maize in hyperarid conditions.

## Introduction

The genetics and phenotypic diversity of *Zea mays* Lam. (maize) has been one of the main topics of study in the attempt to understand the domestication process and the evolutionary history of crops [1,2]. Despite the social, economic and symbolic importance of maize for many prehispanic communities in the Andes, its study has focused on the main areas of maize development, with little attention to its morphogenetic changes outside centers of domestication (México and Central Andes) [3]. Indeed, domestication and the morphogenetic modifications involved are difficult processes to evaluate in the absence of fossil record and plants macro remains [4].

Traditionally, archaeologist describe domestication as the transformation of wild species into a domestic descendant [5]. The latter would present characteristics such as reproductive isolation, the human dependence of the plant for its survival or variations of its grain size, shape, physiological and genetics that differentiate it from its predecessor [6]. However, a more comprehensive and flexible idea of domestication considers this process as a continuum of changes in plants species caused by the interaction between human groups and their environments. Therefore, changes such as increased/decreased in size’s seed and fruits are not always instantaneous or final stages of people-plants interaction [6,7]. Instead, we considered domestication as a nonlinear development, including several attempts to produce desirable transformation in plant resources. Consequently, the archaeobotanical record exhibits many intermediate stages of plants remains.

Also, modifications suffered by plants under human selection are not commonly visible in the archaeological evidence due to preservation conditions, restricting our understanding of the anthropic role in the evolutionary history of plants. However, in Tarapacá region, Atacama Desert (18–21° S), prehispanic settlements (ca. 2500–400 yr BP) displayed an extensive agriculture with abundant and well-preserved maize macro-remains, due to the hyperarid conditions of this region [8,9,10,11]. Also, prehispanic farmers cultivated maize outside the traditional nuclei of diversification and domestication, under arid conditions and in the context of political and social heterogeneous societies. Therefore, we had the extraordinary opportunity to examine the morphogenetic changes suffered by maize in a 2000 years’ time span in the most extreme environmental conditions to produce cereal crops.

### Maize production in the Atacama Desert

**Fig 1:**
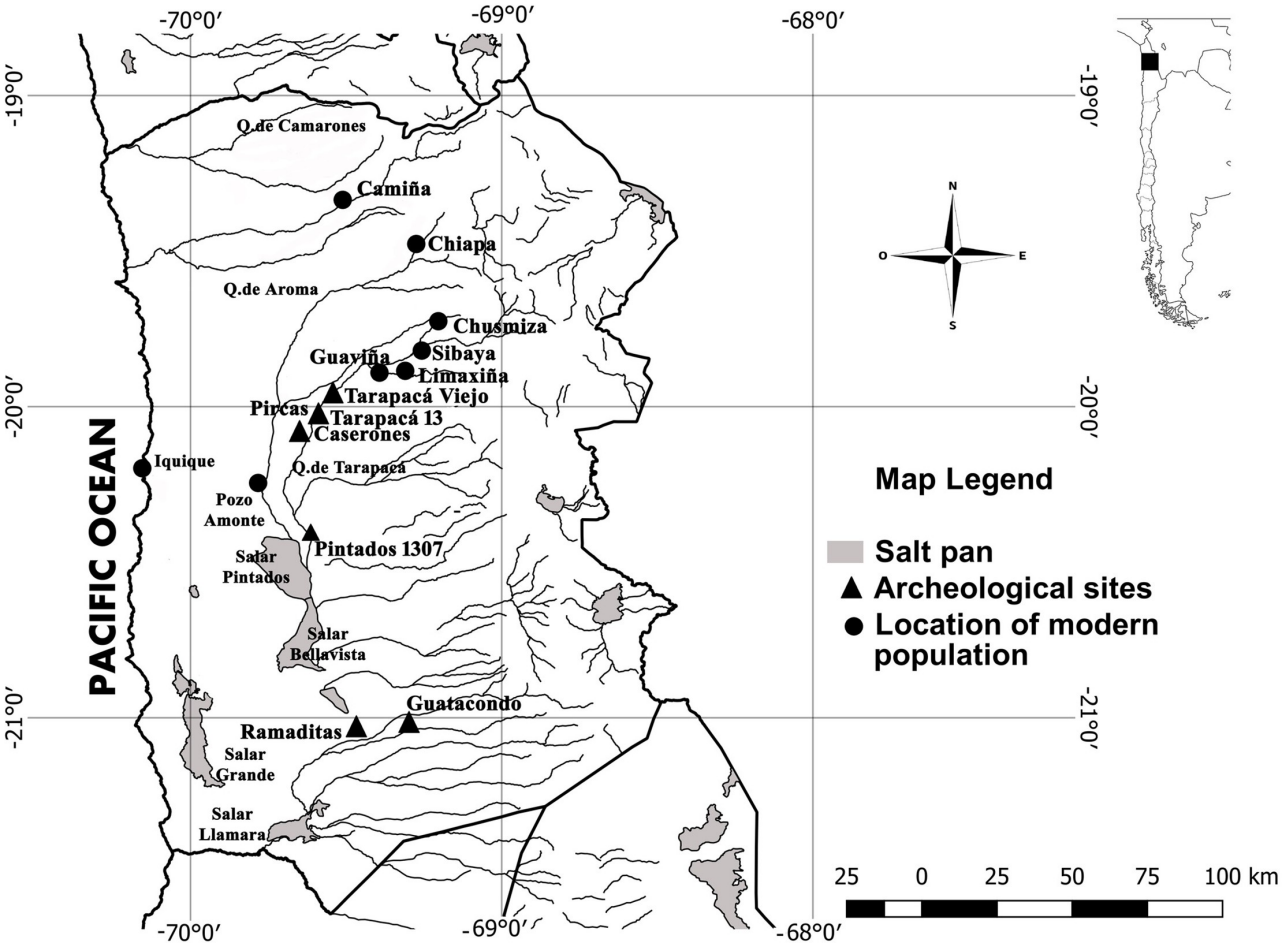
Tarapacá region map. Black triangles indicate archaeological sites dated between Formative Period (ca. 1500–500 yr BP) to Late Period (600–400 yr BP). Black dots indicate modern populations of maize used in this study.

The Atacama Desert, located in northern part of Chile, is considered a unique hyperarid environment with less than <1 mm of annual precipitation. Therefore, prehispanic communities obtained water from groundwater supplied and intermittent runoff water [12]. During prehispanic times this allowed the implementation of extensive agricultural systems in the Tarapacá region and several hectares of Algarrobo forests (*Prosopis* spp.) [13,14,15,16]. Thus, during the Formative Period (*ca*. 3000–1000 yr BP) the emergence of maize agriculture in this region, was concomitant with the development of complex settlements, such as Aldea de Guatacondo (2290–1890 Cal. yr BP), Ramaditas (2340–1870 Cal. yr P), Pircas (2320–1420 Cal. yr BP), Pintados 1307 (1427–1568 Cal.yr BP) and Caserones (1880–1080 Cal. yr BP) [17,18,19,20,21] (Fig 1).

Based on botanical macro remains found in the domestic context of the sites mentioned, social and economic system was partially sustained through the collection of Algarrobo seeds. Also, and intensive agriculture of maize, squash, and quinoa [11] allowed the inhabitants of Tarapacá to exchange several resources such as maize, Algarrobo seeds, cotton and wood with coastal populations [19,20]. However, at the beginning of the Formative Period, maize evidence only represents 3% of macro-remains, and isotopes analysis on human remains indicates a gradual transition to crops consumption [11,22].

After a few centuries, during the Late Intermediate Period (*ca*. 1000–600 yr BP), most archaeological sites situated in the lower part of the Atacama Desert were abandoned, and people moved to higher altitudes into Camiña, Chiapa, Mamiña and Tarapacá valleys [23, 24]. Only a few settlements such as Caserones, Tarapacá 13 (930–490 Cal. yr BP) and Tarapacá Viejo (662–350 Cal. yr BP) endured in the lower parts of the Atacama Desert, developing intensive agriculture that not only modified the desert landscape with several agricultural fields but also modified crops themselves. As a result, cobs and kernels from the Caserones site increased their size during the transition from the Late Formative to the Late Intermediate period [25]. These changes occurred several centuries after the introduction of maize in the region suggesting that human selection was carried out by tarapacá farmers during these periods. However, there is no evidence whether this modification occurred during the early introduction of maize in Tarapacá due to induced changes in the genetic variability of maize or if it was a gradual or sudden change.

Indeed, there is a limited record of the genetic and phenotypic diversity for modern maize in the region and practically no studies for prehispanic samples. In the 1990’s, there were only nine landraces reported for Tarapacá [26, 27]. For prehispanic times, only one cob (dated to 1500 ± 50 yr BP), has been genetic analyzed indicating the introduction of the GA_4_TA allele from the eastern slope of the Andes (sequenced from the *ADH2* gene) [28]. Despite the scarce information for Tarapacá’s maize, Andean landraces exhibit reduced allelic frequencies compared to the Mexico or Caribbean maize. This characteristic has been considered the result of a bottleneck and a founder effects [29,30,31] or the consequences of directional selection by selective breeding [32]. As an effect of one or all of the factors above, there is a broad consensus that both genetic and phenotypic diversity tend to decrease in traditional agricultural systems [33,34].

Hence, according to the archaeological and ecological data, the primary goal of this research is to study the morphogenetic changes in Tarapacá’s maize by comparing its genetic and phenotypic diversity from prehispanic to modern times. Thus, our questions are: How has Tarapaca's maize changed over time? Do these changes reflect the introductions of new varieties or do they indicate an *in situ* domestication processes in an arid environment? Moreover, do the changes previously observed in maize´s size reflect a single change event or a gradual process? Given the artificial selection performed by Tarapacá farmers on crops and the intensive agriculture practiced in Tarapacá since prehispanic times, we expect prehispanic maize to show greater genetic and phenotypic diversity compared to later and modern specimens. Given the hyperarid environmental conditions and the isolated cultural situation of the Tarapacá region, low genetic and phenotypic variability is expected in this time span of 2000 years.

## Material and methods

We collected a total of 95 ears from three traditional landraces, corresponding to six modern populations, distributed in five different locations of Camiña and Tarapacá valleys. Locations are situated in an altitudinal gradient ranging from 2400 to 3300 meters above sea level (m.a.s.l).(Table 1).

**Table 1:** Sample material of traditional landraces.

Population ID for landraces	Valley	Collection Sites	LAT/LONG.	Elevation of the cultivar(m.a.s.l)	Sample size	Material collected
Chusmiza 1	Tarapacá	Chusmiza	19° 41’- 69°11’	3360	17	Ear and husk
Limaxiña 2	Tarapacá	Limaxiña	19° 47’- 69°10’	3150	19	Ear and husk
Huaviña 3	Tarapacá	Huaviña	19° 47’- 69° 13’	2470	10	Ear
Camiña 4	Camiña	Camiña	19° 18’-69° 25’	2496	15	Ear and husk
Camiña Alto 5	Camiña	Camiña alto	19° 18’-69° 24’	2615	19	Ear
Camiña 6	Camiña	Camiña	19° 18’-69° 25’	2414	15	Ear and husk

Landraces samples and type of material studied. Pop, modern population; Valley, valleys of provenance of modern samples; Location, locations of modern samples; LAT/LONG, latitude and longitude coordinates of modern locations; Elevation, meters above sea level of each location; N, number of specimens (ears) collected for each population; Type, vegetative part collected for each population.

Modern ears and husks were collected randomly during May 2014. The collected husks were preserved with silica gel, immediately after collection, while ears were maintained in an isolated room at 10°C degrees until all the morphological and genetic analysis were performed. Today they are kept at Pontificia Univesidad Católica de Chile while this research continues.

We morphologically analyzed a total of 123 archaeological cobs and 151 archaeological kernels (archaeological samples). These samples were obtained from the archaeological sites of Pircas (2320–1420 Cal. yr BP), Pintados 1307 (1427–1568 Cal. yr BP), Tarapacá 13 (930–490 Cal. yr BP) and Tarapacá Viejo (662–350 Cal. yr BP) during the archaeological excavations of their domestic contexts (Table 2). These range times summarize 28 radiocarbon dates (14C) made over organic material recovered from domestic context of habitational enclosures. All the material dated belonged to the same enclosure and stratigraphic level of cobs and kernels use in this study. In Tarapacá Viejo one date was made directly over a cob dated cal. 310 yr BP (Beta 269052) and in Tarapacá 13 six dates were made directly over cobs. These six cobs dated between cal. 835 to 645 yr BP (AMS dating code 58344-45-46-50-55).

**Table 2:** General information of archeological samples.

Location	Collection Sites	Archaeological Populations ID	Sites elevation (m.a.s.l)	Cultural Periods	Time span of archaeological sites (yr. BP)	Cobs sample sizes	Kernels sample sizes
Tarapacá, Chile	Pircas	Pircas	1312	Early Formative Period	2320–1420	15	1
Tarapacá	Pintados 1307	Pintados 1307	1012	Late Formative Period	1427–1568	0	6
Tarapacá, Chile	Tarapacá 13	Tr-13	1310	Late Intermediate Period	930–490	26	32
Tarapacá, Chile	Tarapacá Viejo	Tr Viejo	1425	Late Intermediate Period and Late Formative Period	662–350	82	112

General information of archeological samples. Location, locations of archeological sites; Collection Sites, archeological sites of sample provenance (Pintados 1307, Pircas, Tarapacá 13 and Tarapacá Viejo); Archaeological Population ID, Site elevation, meters above sea level of archaeological sites; Cultural Period, time/cultural period associated according to the cultural sequence of the South- Central Andes; Time span, is established by calibrated 14C radiocarbon dates; Cobs saple size is the number of cobs analyzed; Kernels sample size is the number of kernels analyzed for each archeological population.

The archaeological samples have been collected during several years of archaeological excavation under the project FONDECYT 1130279 and FONDECYT 7060165. All necessary permits were obtained for the described study, which complied with all relevant regulations. Archaeological samples are deposited permanently in the Anthropology Department of Universidad de Chile (macro botanical section-provenance site).

A total of 13 traits were measured and compared in cobs and six traits in kernels between archaeological and modern samples (S1 Table). Modern cobs and kernels were dehydrated, to partially replicate the conditions found in the archaeological samples, allowing us to compare both samples. To determine shape on cobs and kernels, we measured the Shape factor (S) and Feret diameter (DF) using SIGMA SCAN PRO 5.0. To establish the variability of morphological traits we performed Principal Component Analysis (PCA) performed in PAST 3.4 [35]. Linear Discriminant Analysis (LDA) and ANOSIM test were performed to determine the probability of correctly attributing a given kernel and cob to the different clusters present. Also, Permutational Multivariate Analysis of Variance (PERMANOVA) was performed to determine the statistical significance of morphological diversity between archaeological and modern samples.

Eight microsatellite markers or simple sequence repeats (SSR) were analyzed (S2 Table) an M13 (CACGACGTTGTAAAACGAC) tail was added to label amplicons for visualization on the capillary sequencer [36]. We select SSR primers according to their polymorphism index and their chromosomal locations in order to cover a wide range of chromosomal bin (Phi029, Phi034, Phi 056, Phi059, Phi063, Phi075, Phi127 and umc1332). Because archaeological samples commonly exhibit degraded DNA, we choose SSR of small size [30,31]. The SSR were obtained in MaizeGDB (www.maizegdb.org).

DNA extraction was carried out on husks and roots in 95 modern samples (named as Chusmiza 1, Limaxiña 2, Huaviña 3, Camiña 4, Camiña Alto 5, Camiña 6), over 21 archaeological kernels (named as archaeological population) and six fragments of archaeological husk (named as HTr13-1 to HTr13-6) following a standard protocol based on CTAB buffer Husk fragments were used as an extra negative control since given its state of conservation we assumed that it did not contain DNA. Ancient DNA (aDNA) and modern DNA (mDNA) extractions were performed in a clean laboratory. The extractions were performed one year apart from each other avoiding any possible contamination.

To check the integrity of DNA in archaeological samples, we tested DNA extractions using NANODROP and QUBIT to establish the DNA double helix concentration. (S3 Table). Additionally, we checked the aDNA concentration post amplification through an agarose gel. All the specimen used, obtained more than 15 ng/μl.

We analyzed SSR using Peak Scanner Software 2.0 (Applied Biosystems 2012) and Genious v.5.0 and checked for null alleles in Micro Checker v.2.2.3.

Amplification of ancient and modern DNA samples was carried out using Polymerase Chain Reaction (PCR). The PCR products were checked in agarose under electrophoresis and visualized under UV light.Fragment analysis by capillary electrophoresis was performed in the sequencing laboratory of the Pontificia Universidad Católica de Chile (S4 Table).

Genetic diversity was estimated by allelic frequency rates, the average number of alleles per locus, observed heterozygosity (Ho) and expected heterozygosity (He). Differences between populations were estimated by fixation index F_ST_ in GenAlEx 6.5 [37]. A hierarchical cluster analysis with F_ST_ values and a genetic structure population analysis using STRUCTURE v2.3.4 [38] were performed to establish the genetic distance between groups. Structure analysis uses a Bayesian approach to identify the number of genetic clusters (K) and assign probabilistically each individual to these clusters without a priori knowledge of putative populations. Ten independent runs were carried out for K ranging from 1 to 7 using a Markov Chain Monte Carlo run length of 100,000, a burn-in of 100,000 and an admixture ancestry model assuming correlated allele frequencies. To determinate the most likely number of clusters (K) we used the rate of change in Ln P(D) (the log probability of data) between successive K values [39].

During the collection of modern specimens, we interviewed nine local farmers to gather information about the following items: 1) provenance of seeds, 2) vernacular name of the variety, 3) age of maize sample collected, 4) sowing and harvesting times, 5) soil and water requirements, 6) use of fertilizer 7) hybridized or not hybridized 8) general characteristics of the maize varieties. An interview form was designed, and questions followed an open structure interview. These landraces were identified according to the parameters used previously descriptions [26, 27].

## Results

### Phenotypic comparison between archaeological and modern samples

**Fig 2:**
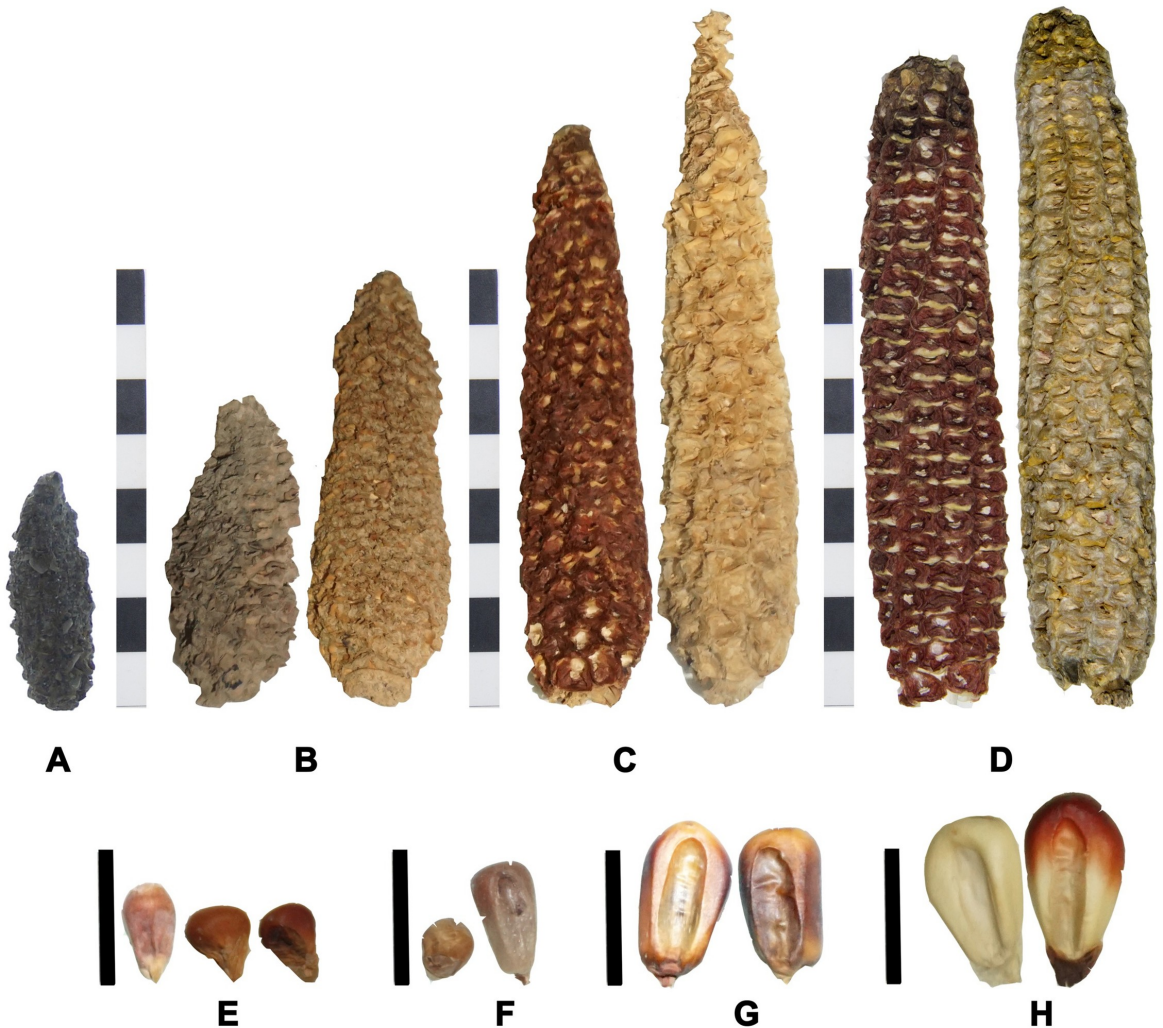
Cobs and kernels from archaeological and modern populations. A) cobs from Pircas sites, B) cobs from Tarapacá 13, C) cobs from Tarapacá Viejo, D) modern cobs from Camiña and Tarapacá respectively. E) kernels from Pircas and Pintados 1307, F) kernels from Tarapacá 13, G) kernels from Tarapacá 49, H) modern kernels from Camiña and Tarapacá, respectively. Scale bar cobs = 8 cm, scale bar kernels = 1cm.

Our results suggest a progressive increase in size for both cobs and kernels, between archaeological and modern samples (Fig 2). The earliest cobs samples reach an average of 5.86 mm length while the latest cobs show an average of 109 mm length. Archaeological kernels reached an average length of 10.64 mm while modern ones are 14.78 mm (S1 Table).

**Fig 3:**
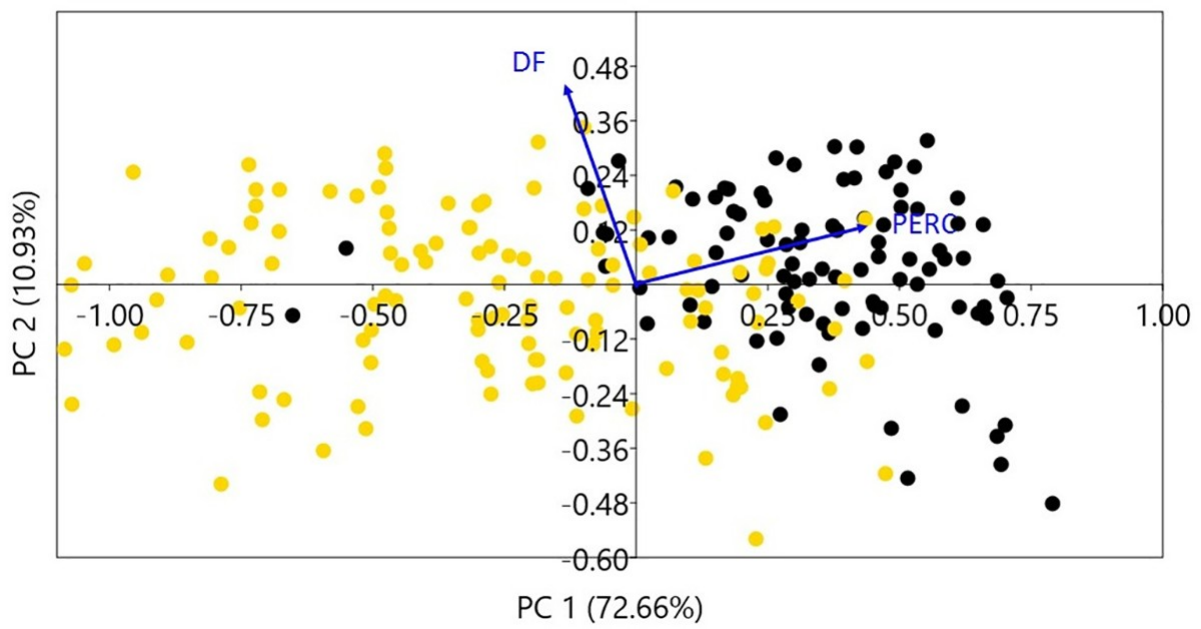
PCA of archaeological and modern cobs for 10 traits. First two PCA axes explain 83.59% of the total variance. Yellow dots represent archaeological cobs. Black dots represent modern cobs. The PC1 axis associated with the perimeter variable separates in two groups: modern and archaeological cobs. PERC = Perimeter of cobs; DF = Feret diameter.

PCA analysis presents clear segregation between archaeological and modern samples. The first two axes of the PCA of archaeological and modern cobs accumulate 83.59% of the total variance; PC1 explained 72.66% and PC2 10.93%. PC1 is mainly explained by perimeter (loading = 0.658) and PC2 Feret diameter (loading = 0.624). Prehispanic cobs are consistently smaller in their perimeter and separated from modern cobs. Only 14 cobs reached current size; all these specimens belong to the Late Period under the Inca state presence (Fig 3).

**Fig 4:**
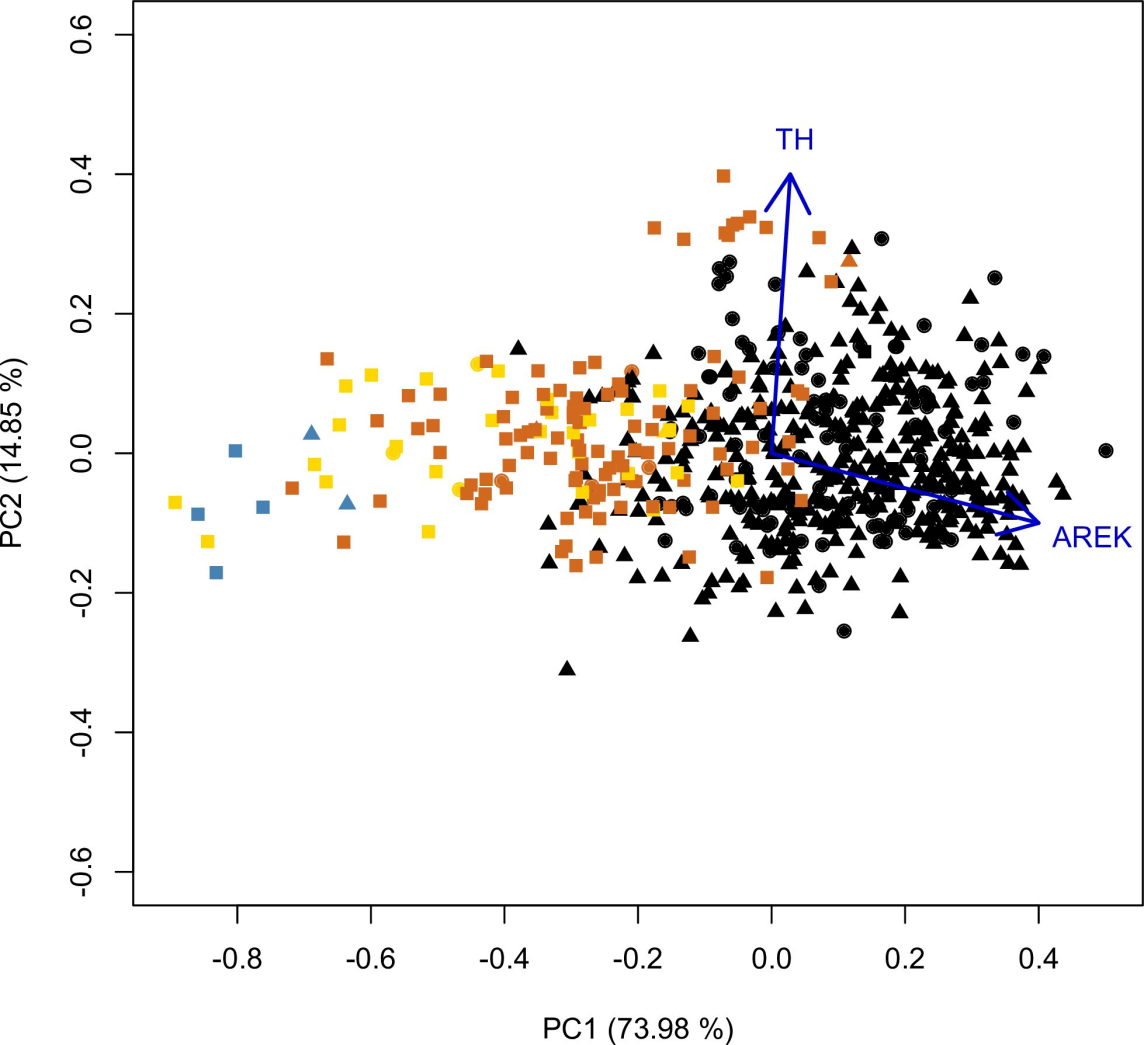
PCA of archaeological and modern kernels for five traits. First two PCA axes accumulate 88.83% of the total variance. Blue dots represent archaeological kernels from Pintados 1307, yellow dots represent archaeological kernels from Tarapacá 13, orange dots represent archaeological kernels from Tarapacá Viejo, and black dots represent modern kernels. Kernels from Pintados 1307 (Formative Period) are grouped with Tarapacá 13 kernels (Late Intermediate Period-Late Period), while Tarapacá Viejo (Late Period) and modern kernel specimens are distant from the previous ones. AREK = Area of the kernel; TH = Thickness., Circles correspond to individuals assigned to genetic cluster 1 in Structure analysis, triangles correspond to individuals assigned to genetic cluster 2 and squares correspond to individuals with no genetic information.

PCA analysis over kernels indicates that the first two axes of the PCA of archaeological and modern kernels accumulate 88.83% of the total variance; PC1 explained 73.98% and PC2 explained 14.85% PC1 is mainly explained by the kernel area (load = 0.759) and PC2 mainly by the thickness (loading = 0.977). Pintados 1037 (Late Formative) kernels are segregated from the rest, as they are round and smaller (blue dots). Kernels from Tarapacá 13 (yellow dots) are smaller than Tarapacá Viejo (orange dots), showing a progressive increase of size from the Late Intermediate Period into the Late Period. Kernels from Tarapacá Viejo are the largest of the archaeological samples, and some specimens even reached modern size. Finally, modern kernels (black dots) are separated from all the rest, distributed in the right half of the biplot (Fig 4).

**Fig 5:**
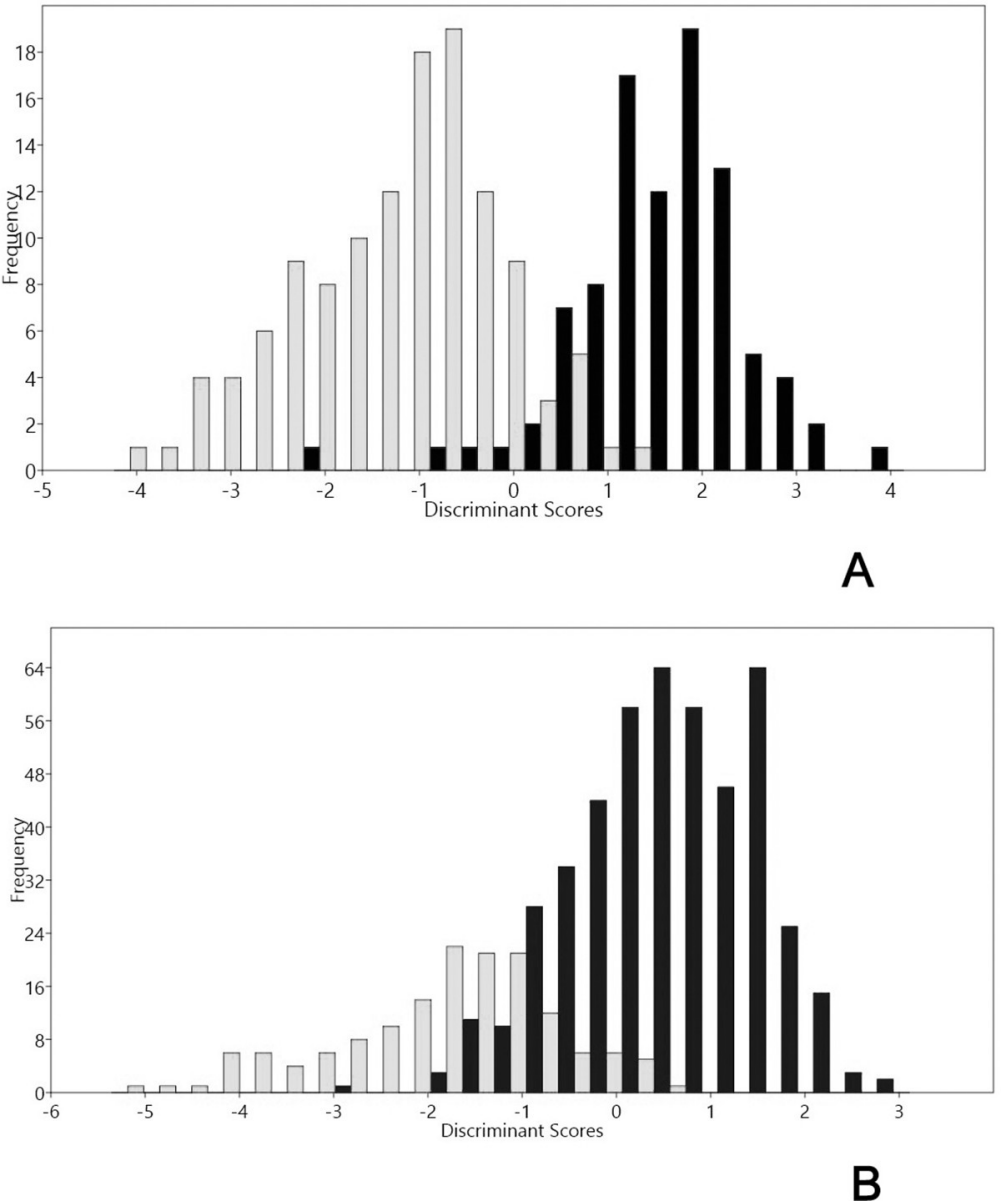
A) Histogram of discriminant scores for 10 traits in cobs and, B) five traits in kernels. Light bars show archaeological cobs and kernels; black bars show modern cobs and kernels. Two separated groups are present: modern and archaeological cobs and kernels.

Accordance with LDA analysis, archaeological and modern cobs discriminate into two groups (Group 1 modern; Group 2 archaeological). Group 1 contains 96% of individuals, and Group 2 contains 93% of individuals. Area variable (loading = -0.15804) explains LD 1. ANOSIM test performed over this two groups indicates a statistic R = 0.4393 (p<0.001). LDA on kernels discriminates two groups (Group 1: modern; Group 2 archaeological samples). The correct allocation percentage for Group 1 is 87%; meanwhile, the correct allocation percentage for Group 2 is 88%. The primary variable for LD1 is Area (loading = 0.1303). ANOSIM test performed over this two groups indicates a statistic R = 0.5593 (p<0.001) (Fig 5).

### Genetic comparison between archaeological and modern samples

We obtained appropriated DNA concentrations for twelve archaeological kernels that tested positive for amplification. We did not obtain DNA from archaeological husks as it was predicted, indicating that positive results for kernels are not due to any modern contamination (S3 Table).

Archaeological kernels showed the same allele amplification as modern samples, except allele 158 (Phi 029), allele 153 (umc1332), allele 288 and 258 (phi075) present as private alleles and not found in our modern samples (S5, S6 and S7 Table). The two kernels dating to the Late Formative Period showed the same range of amplification as the Late Intermediate and Late Period samples. The number of different alleles in archaeological samples was 2.8, and the number of effective alleles per locus was 2.3. Ho/He showed an excess of homozygotes under the assumption of Hardy–Weinberg equilibrium (average 0.164 [Ho] and 0.479 [He]). The average number of different alleles for the six modern populations was 3.6. The mean number of alleles per locus was 5.1, with a minimum of 4 and a maximum of 9. The ratio of heterozygosity (Ho) over expected heterozygosity (He) showed an excess of homozygotes under the assumption of Hardy–Weinberg (average 0.413 [Ho] and average 0.521 [He]), except for three loci (Phi029, umc1332, and Phi034) (Table 3).

**Table 3:** Genetic diversity in archaeological and modern samples.

Population	Avg. of individual	He	Ho	Alleles per locus
Modern populations	15.708	0.521	0.413	5.1
Archaeological population (7)	12	0.479	0.164	2.3

Avg. of individual; the average number of individual in each sample; He, the average expected heterozygosity; Ho, the average observed heterozygosity; Alleles per locus, average number of alleles per locus.

**Fig 6:**
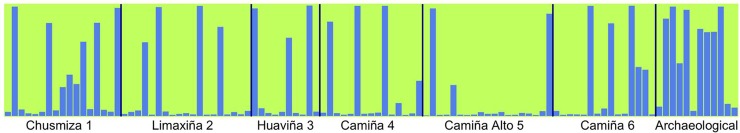
Estimated population structure. Each population assigned in the lower part of the graphic. The graphic is portioned into K = 2 colored segments (green and red segments). The population are separated by black bars and labeled above the figure.

STRUCTURE analysis produced the highest ΔK (6.777) score in *K = 2*, but no clear geographical genetic structure was observed. All populations in Structure analysis show admixture, being population Camiña Alto 5(modern) and the archaeological population more homogenous than the rest (Fig 6).

**Fig 7:**
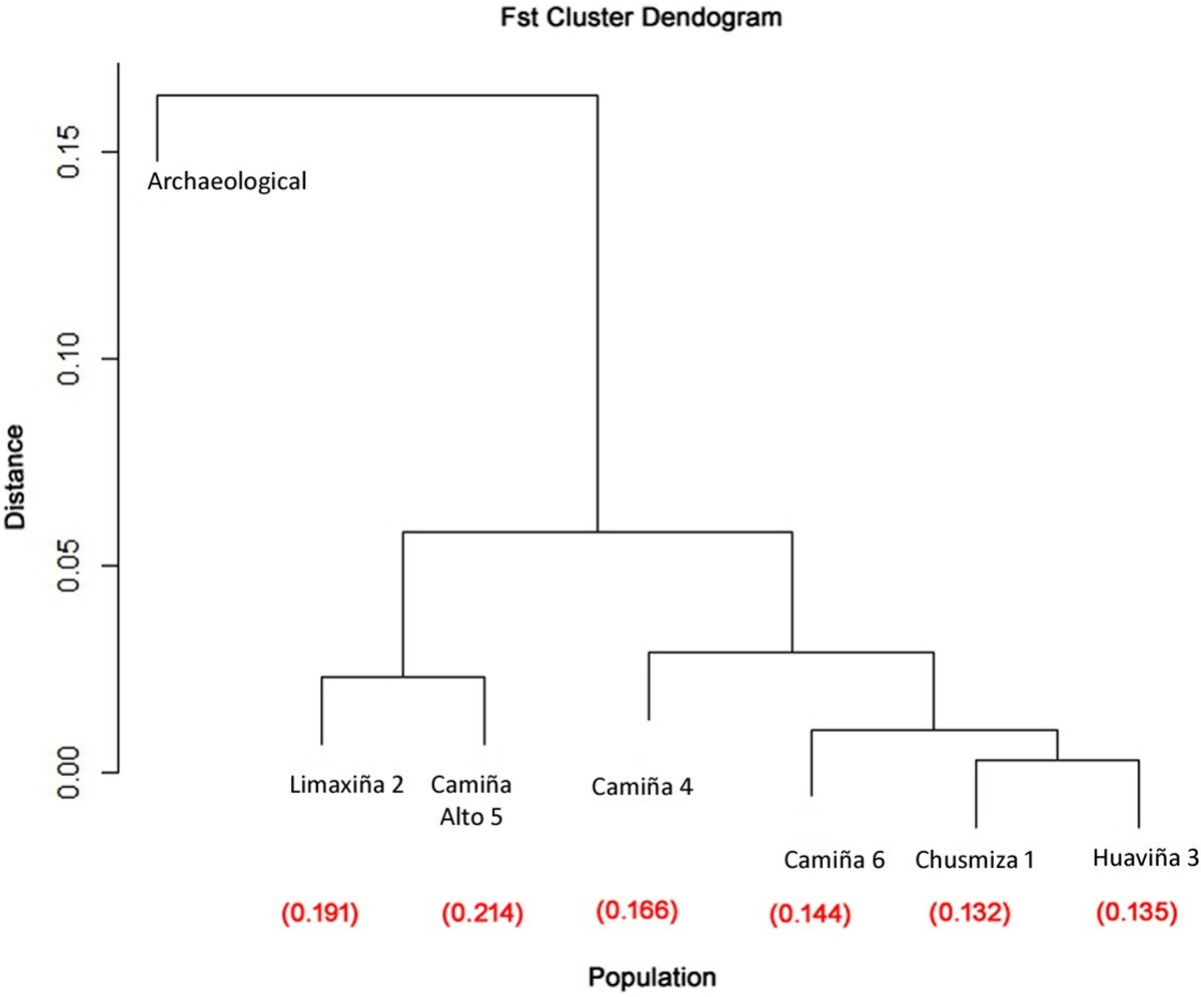
Cluster dendrogram for F_ST_ values for modern and archaeological populations. Three major groups are observed; modern populations Limaxiña 2 and Camiña Alto 5 are grouped in one branch, populations Chusmiza 1, Huaviña 3, Camiña 4 and Camiña 6 are grouped in a second branch, and the archaeological population) is distant from the rest. Fst value between archaeological and modern populations are listed below each population.

The K = 2 is due to the difference observed between modern and the archaeological population, reflecting a chronological distance between samples. This result is consistent with clustering based on genetic distance by Fst values segregating archaeological samples from the rest of the modern populations. As is shown by hierarchical cluster analysis of the F_ST_ values, three significant clusters are displayed. The first cluster includes modern populations Limaxiña 2 and Camiña Alto 5 and the second cluster includes modern populations Chusmiza 1, Huaviña 3, Camiña 4 and Camiña 6, while the archaeological sample (population 7) is distant from the rest of the clusters (Fig 7).

AMOVA analysis indicates that 23% of the molecular variance is explained among regions, 75% within populations and 2% among populations. This percentage among regions is consistent with the results shown by Structure and F_ST_ values.

### Agronomic information of modern and archaeological populations

The qualitative information gathered and summarized in Table 4 shows six vernacular names given to three traditional landraces collected (*Harinoso Tarapaqueño*, *Capia Chileno Chico*, *and Chulpi)*. These landraces correspond to soft endosperm maize and are differentiated by farmers according to the color and taste of kernel, and the shape of the cob. These varieties are all open pollinated.

**Table 4:** Summarize agronomic information.

Charac.	PROV	Landraces names	Vernacular names	Sample age at time of collection	SH	WC	F	L. PROV
Name of populations								
Chusmiza 1	L/F	*Capio Chileno chico*	*Choclo blanco*	0	Ag-Ap	SW	P	Chusmiza
Limaxiña 2	L	*Capio Chileno*	*Choclo colorado or Choclo morado*	0	Dc-My	SW	NA	Limaxiña
Huaviña 3	L/F	*Harinoso Tarapaqueño*	*Choclo blanco*	1	Ag-Fe	NA	P	Huaviña
Camiña 4	L	Harinoso Tarapaqueño	Choclo blanco	4	Ju-Dc	NA	P	Camiña
Camiña Alto 5	F	*Chulpi* *Capio Chileno chico*	*Chulpi*, *Capio*, *Choclo blanco*, *Choclo colorado*	2	Nv-Ma	NA	P	Camiña
Camiña 6	L	*Harinoso Tarapaqueño*	*Choclo de Camiña*	0	Ju-Jn	S	P	Camiña

Summary of agronomical and anthropological information concerning characteristics of maize used for this study. PROV, provenance of seeds L = local, F = foreign; VN, vernacular name of the variety; SA, indicates the age sample in months at the moment of collection where 0 = collected directly from the plant; SH, sowing and harvesting times (My-J-Ju-Ag autumn-winter, Nv-Dc-Jn-Fb-Ma-Ap spring-summer); WC characteristics of water S = salty, SW = Sweet; F, use of fertilizer presence = P, Absence = A, L. PROV, locality associated with maize sample; PA, Maize population associated. NOA = no information available.

Sowing and harvesting times tend to vary from five to nine months. Farmers mentioned that almost all maize varieties need sweet water; only population from Camiña 6 was resistant to salty water. Seven farmers indicated the use of urea and potassium nitrate as fertilizer; in one case (population Limaxiña 2) this information was not available. Other qualitative information obtained during our visit, was that Camiña maize has faster maturation time, due to longer sun exposure than Chusmiza or Limaxiña maize, which are situated above 3000 m.a.s.l. Farmers from Huaviña affirm that sun exposure time affects maturation and the size of the ears, therefore Chusmiza maize is smaller than Huaviña, according to their appreciation. This observation was mentioned to us only once and is not corroborated by our analysis.

According to previous studies [26, 27] Harinoso Tarapaqueño has medium, thick, conical ears with rounded tip completely covered with grains, with regular and abundant rows. The grains are floury, long, dentate and colorless, variegated or reddish pericarp. Plants can reach heights from 2.30 m to 4.50 m [26].In our observation Harinoso Tarapaqueño plants reached 2.5 m tall.

Chulpi is a landrace describe to possess medium ears size with rounded or bell shape with abundant irregular rows. Grains are a floury type and elongated [26]. Plants of these varieties can reach from 1.31 m to 2.7 m [26]. In our observation plants reached a 1.6 m tall.

**Fig 8:**
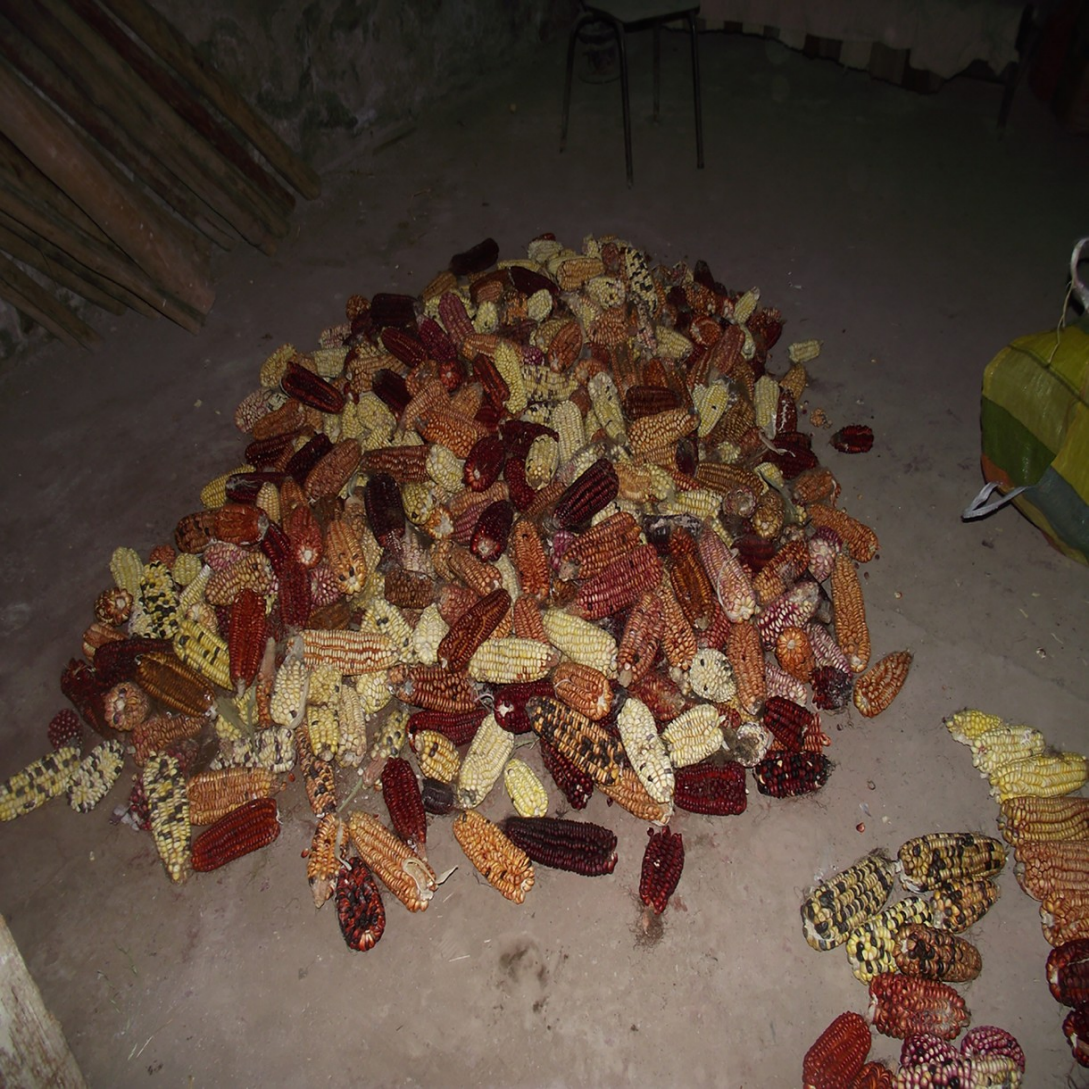
Several ears of Chulpi, Harinoso Tarapaqueño and Capio Chileno from Camiña valley.

Capio Chileno Chico has short and medium-size ears, rounded or conical, with irregular rows of kernels. Grains are floury types, with a light indentation at the crown, purple and speckled aleurone. Some grains are white and yellow [26] (Fig 8).

Farmers indicated that the original seed of populations Chusmiza 1, Huaviña 3 were from nearby villages, situated in the same basin of Tarapacá. In the case of Camiña Alto 5 maize seed presumably came from an entirely different valley (Chiapa village) located at higher altitude in Tarapacá region. Thus, farmers confirmed seed exchange is intentionally and consciously performed in present time, between Tarapacá and Camiña communities. Finally, two farmers told that they selected ‘larger grains’ and ‘cobs with regular disposed rows’ (in reference to Chusmiza 1 and Huaviña 3 population respectively).

**Fig 9:**
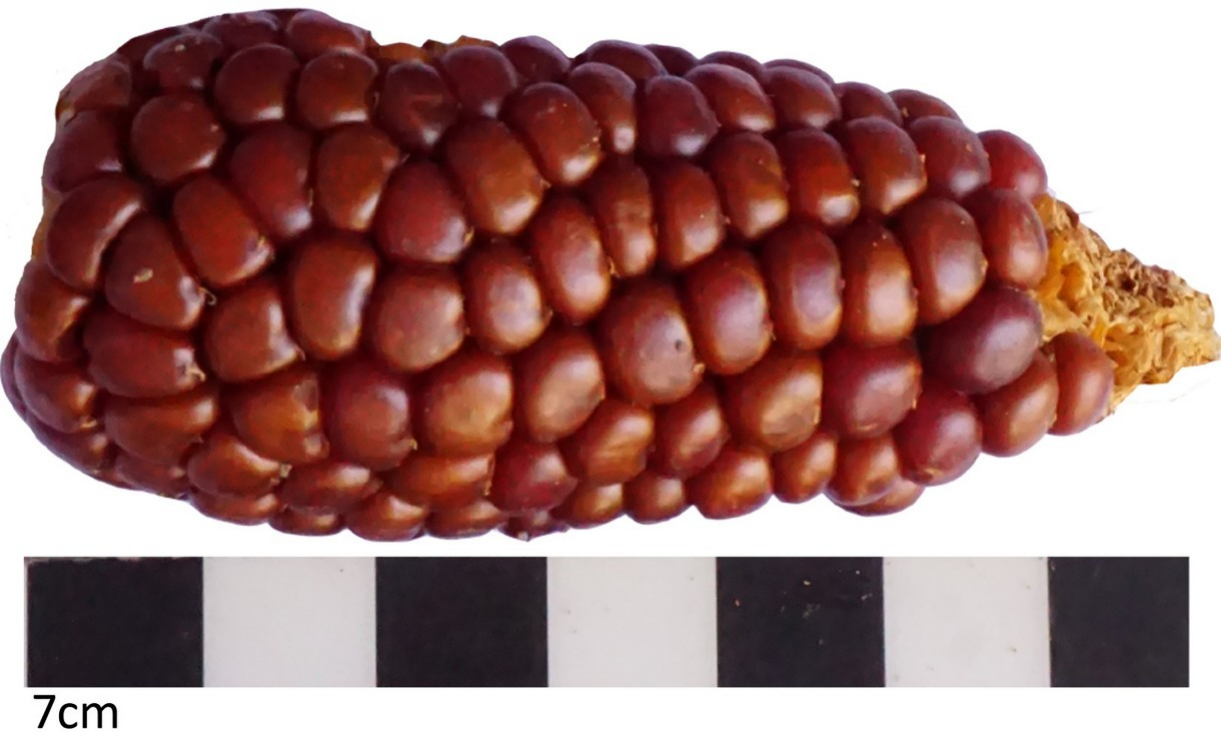
Archeological popcorn ear from Tarapacá.

The archaeological samples correspond to two types of kernels. Pintados 1307 have small rounded, red to brown color pericarp, and a white endosperm kernels.This variety is a popcorn type and several kernels appear popped Kernels found in Tarapacá 13 and Tarapacá Viejo have a soft endosperm similar to modern specimen of Tarapacá. The color of the pericarp varies from light white and yellow to dark brown and violet. The endosperm is white. Most kernels are plain and have diamond form with a semicircle crown. (Fig 9).

## Discussion

Significant phenotypic and genetic differences are recognized between archaeological and modern maize in Tarapacá suggesting that Tarapacá maize changed over time. The comparison of morphological traits between archaeological and modern samples is consistent with differences in cobs size and kernels size. In contrast, we observed no genetic differences but a progressive increase in kernels size within the archaeological samples during the Late Intermediate Period and Late Period. Accordingly, cobs and kernels from Tarapacá Viejo (Late Period/Inca period) reached for the first time in Tarapacá history the size of modern maize.

Modern and archaeological populations exhibited low genetic diversity parameters compared to adjacent areas. Previous analysis of 235 plants of Andean provenience showed an average of 12.4 alleles per locus and an observed heterozygosity value of 0.706 [2]. Similarly, 176 modern Andean specimens indicated an average of 6.1 alleles per locus [30]. Although this could due to the smaller size of our sample and the lower quantity of SSR used, maize from Tarapacá may show a founder effect as it was suggested for the Andean Group [28, 31]. Concordantly, Fst values segregated the archaeological sample from the modern sample, indicating an isolation process and therefore the generation of genetic differentiation between samples. According to this, we propose that several years of human selection would have decreased and change the allele frequencies.

Although genetic drift has acted as main force in genetic structure of modern hybridized maize due to the reduce effective population size [40], it also has been demonstrated that heterozygosity decreases more than expected at the genome level as whole in population under artifictial selection [34,40].The excess of homozygotes in both samples is concordant with an inbreeding process that supports the selection hypothesis [41]. Similarly, homozygous excess as a result of inbreeding has also been reported for primitive landraces of maize [42], other crops such as rice [43] and domesticated animals [44]. Indeed, Andean farmers are constantly selecting characters like the size and color and shape of ears and kernels, disposition of kernels and taste, among others, to fulfilled cultural expectations [45].

A second factor are the environmental conditions such as altitude (from 2400 to 3300 m.a.s.l), low nutrition soils, low daytime sunlight and temperature gradients. In these circumstances only, a few seeds could have adapted to the Tarapacá hyper-arid environment, consequently decreasing genetic diversity parameters and generating the excess of homozygous. But these natural conditions might have been mediated through human preferences and observations. Farmers selected seed from plants that exhibited improved yields and drought resistance.

As well, these genetics parameters respond to the social and agronomic history of Tarapacá. Unlike other areas of South Central Andes, the Tarapacá region shows relative cultural isolation for 600 years (ca. 1500–900 yBP) with limited cultural connection with the state of Tiwanaku [19, 21]. This social dynamic probably restricted seed's exchange with neighboring areas.

The genetic results indicate that archaeological samples amplified loci for all SSR identical to those observed in modern samples, except for four private alleles: 158 (phi029), allele 153 (umc1332), allele 258 (phi075) and allele 288 (phi075), only present in the archaeological samples. Similar studies of archaeological specimens of maize in northwestern Argentina highlighted the lack of genetic variation, exhibiting a single allelic variant identical in size to modern populations for allele 112 (Phi 127), allele 154 (Phi 029) and allele 157 (Phi 059) [31]. Compared to previous analysis, specimens of inbreed lines from Argentina amplified for allele 158 (Phi029) [46], and from North Western Argentina [31]. Allele 258 (Phi075) has been reported for the Andean Group maize in primitive and historical landraces of the highlands of Perú [30]. We did not find reports for the presence of allele 153 (umc1332) or allele 288 (Phi075). For the rest of the archaeological sample allele 167 (Phi029), allele 176 (Phi 029), allele 159 (Phi 034), allele 259 (Phi 056), allele 262(Phi 056), allele 268 (Phi 056) and allele 192 (Phi063), are present at the Andean Group maize of Perú [30]. For the rest of the alleles, we found no match with other researches (for a comparison of SSR see S6 Table).

Despite the reduced number of SSR used in this study, this initial evidence suggests an introduction of some genetic pool from Argentina and Perú. Likewise, one exemplary from Tarapacá contained the GA_4_TA allele from the eastern slope of the Andes [28]. Also, in Caserones we registered foreign species such as *Anadenanthera colubrina* (*cebil*) and *Aspidosperma quebracho-blanco (quebracho)* [11], which are today nearest distributed in Chaco region (Argentina). The genetic data and the archaeobotanical remains are starting to suggest that Northwestern of Argentina and Chaco region are suitable candidates for the origin of Tarapacá maize. This hypothesis will be tested in the future by adding more markers and archaeological samples.

In summary, our phenotypic and genetic data propose a) loss of alleles and alteration of allele frequencies as a result of a selection process during pre-Hispanic times or b) an introduction of a new genetic pool in Tarapacá.

During the Formative Period, adequate environmental conditions were present for the production of maize in Tarapacá. The pollen record from rodent middens and plant macrofossils from latest Holocene leaf litter deposits in the southern portion of Tarapacá region indicates an increase in moisture between 2,500–2040, 1615–1350 and 1050–680 years cal BP. These conditions would have allowed the recharge of the aquifers and the presence of permanent runoff water [12, 47, 48, 49]. Accordantly, the plant remains of paleo rodents middens support the existence of greater humidity in the *altiplano* (> 3000 m.a.s.l) making possible extensive agriculture and allowing the settlement of villages and sites at low altitudes (1100–1300 meters) [50].

However, the limited presence of maize during the Early Formative Period (2500–2000 yr BP) suggests that it was not a significant economic crop at the beginning of agriculture. Accordingly, studies conducted on stable isotope analysis (d13C and d15N) in Tarapaca-40 formative human remains, suggest that maize would not have been relevant in the diet of people during early times [22], but probably its presence is due to ritual activities or other extraordinary functions [51]. The low genetic variability observed in modern and archaeological samples corroborates this assumption, indicating a possible late introduction and further development of maize agriculture. Massive production of maize occurred during the Late Formative Period (ca.1700-800 yr BP) constituting more than 90% of the plant resources at the site of Caserones [11].

During the Late Intermediate Period (ca.800-550 yr BP), environmental conditions changed to a drier regime [45]. Accordingly, most settlements during this time are situated at higher altitudes (2200–2600 m.a.s.l) where there is more water available. Only a few settlements, such as Caserones and Tarapacá 13 (1200 m.a.s.l) remained in the lower portion of the basin of Tarapacá. The cultural consequences are that Caserones became a core center of maize production with human selection operating on size [25]. Our results indicate that Tarapacá 13 kernels are distant from previous kernels, progressively increasing their size, maybe as the receptor of Caserones production.

During the Late Period (ca. 550–465 yr BP) humid conditions returned, and Tarapacá Viejo basin was occupied by the Inca state [52,53,54]. It has been suggested the Inca state introduced new resources in areas under its domain, especially in agricultural production [55,56]. Our results contradict this assumption because we observed the same types of kernels (floury varieties) in Tarapacá 13 and Tarapacá Viejo. An earlier agricultural system was well established before the arrival of the Incas in Tarapacá, as shown by Caserones farmers in their effort to enhance maize size. However, some changes did occur under Inca presence. The size of maize's cobs and kernels from Tarapacá Viejo are larger than previous ones reaching the same size as modern specimens. We postulate that Inca strategies focused on increase maize size (specifically kernel size) and the quantity of maize production.

Although we do not know the specific biological mechanism of this size improvement for archaeological specimens, in present times farmers still select the most prominent ears and kernels for sowing. This cultural practice over 200 years would have increased the size of cobs and kernels. Indeed, the high phenotypic diversity of maize is due to transposon activity and the polyploid nature of the maize genome [57]; several genes are known to contribute to phenotypic differences of several traits under selection during domestication or manipulation of the species [58]. Furthermore, changes in a single locus produced significant morphological modifications in maize [59], and experimental conditions proved this is possible to achieve in just a few generations [60]. As an example, Mexican maize cob specimens from the Ocampo Caves (dated from 5550 old to 4400 yr BP) show that cob size increased continuously during the first 2000 years of human selection [61]. The mechanism involves P*bf* and su1 genes, which are related to protein and starch quality. The presence of these genes in Mexican pre-Hispanic cobs suggests that kernel quality and cob size were traits selected early in prehispanic times. Thus we propose that the Inca state did not introduce new landraces of maize in Tarapacá, but reinforced the agricultural system by increasing production, expanding agricultural fields and terraces, channeling water in desert landscapes, focusing on production of floury varieties of maize, and finally increasing cob and kernel size already present in the Atacama Desert.

## Conclusions and archaeological implications

The concept of domestication addressed here goes beyond the notion of a single transition from wild to domesticated plants. Instead, we consider domestication as a complex and continuous process of changes and interaction between human societies and plants. The changes observed in Tarapacá maize (increase in size, low genetic variability, excess of homozygous, and the adaptation to hyperarid conditions are probably caused by the conscious manipulation of Tarapacá farmers that leads to major transformations of crops. If proved, artificial selection by humans is one of the mechanisms to achieve these changes. In the hyperarid conditions of the Atacama Desert, no crops can survive without human assistance. Once ecological conditions changed during historical times, farmers abandoned maize agriculture in the Atacama Desert. In this sense, domestication (*sensu lato*) should also consider successes and failures in the art of plants management. In our case, maize changes responded to concrete and heterogeneous historical situations by politically autonomous groups [62]. Thus, while Caserones may have acted as a nuclear settlement for agricultural development, farmers of Tarapacá 13 might have acted as recipients of this agricultural production. At last the Inca presence continue to increase maize size in a short span time. In summary, the morphogenetic changes observed in maize reaffirm the crucial historical role played by the local substratum of prehispanic and modern communities of Tarapacá in the evolutionary history of maize in the world's most arid desert.

The co-evolution of maize and human societies has reached a point where a puzzle with different pieces is taking shape from the crossing between genetic analysis and the morphology of landraces. To advance in this collective task, we proposed 1) To generate interdisciplinary research: the ancestral knowledge of small-scale farmers, genetics, evolutionary biology, anthropology, and archaeology can provide new insights. 2) To create a comprehensive understanding of the Domestication Syndrome is necessary to establish a historical approach. For that, if possible, we suggest performing analysis of archaeobotanical remains and encompass the longest time as possible. In this respect, hyper-arid systems are proving to be a proper ancient genetic reservoir. And 3) to evaluate small areas, with well-known cultural history, social dynamics and the ecological conditions that surrounded ancient maize agriculture. From our theoretical approach, a small-scale area of analysis and interdisciplinary work could reshape the scenarios of Domestication, allowing us to overcome the reductionist approaches.

## Supporting information

S1 Table Measures in modern and archeological samples. (DOCX)

S2 Table Microsatellites used in this study in archeological and modern kernel samples. (DOCX)

S3 Table aDNA readings and concentrations measured in NANODROP and QUBIT. (DOCX)

S4 Table DNA amplification protocol. (DOCX)

S5 Table DNA size range amplification in modern and archaeological samples. (DOCX)

S6 Table DNa size range comparison with other modern and archaeological Andean maize. (DOCX)

S7 Table SSR data matrix used for this study. (XLSX)
