# Supplementary material for: Genetic and phenotypic diversity in 2000 years old maize (Zea mays L.) samples from the Tarapacá region, Atacama Desert, Chile
Source: PLoS One. 2019 Jan 30;14(1):e0210369. doi: 10.1371/journal.pone.0210369 (PMC6353141; doi:10.1371/journal.pone.0210369)
Supplement: S1 Table — (DOCX) [file pone.0210369.s001.docx]

**S1 Table. Measures in modern and archaeological samples**.

| **Traits** | **Leng** | **U.Dia** | **Me.Dia** | **Ma.Dia** | **Inf.Dia** | **N Row** | **Ker/Row** | **T/kern** | **Wid** | **Thic** | **CI** | **Per** | **Are** | **SF** | **DF** |
| --- | --- | --- | --- | --- | --- | --- | --- | --- | --- | --- | --- | --- | --- | --- | --- |
| **Cob** | **109.5** | **10.33** | **19.69** | **22.26** | **17.25** | **11.98** | **18.94** | **-** | **-** | **-** | **6.48** | **32.35** | **253.07** | **3.22** | **56.17** |
| **Kern** | **14.78** | **-** | **-** | **-** | **-** | **-** | **-** | **-** | **9.68** | **5.88** | **-** | **45.11** | **1.11** | **6.78** | **11.77** |
| **a.Cob** | **58.6** | **9.78** | **-** | **17.65** | **13.83** | **10.53** | **11.66** | **-** | **-** | **-** | **7.24** | **18.92** | **9.67** | **0.36** | **3.39** |
| **a.Kern** | **10.64** | **-** | **-** | **-** | **-** | **-** | **-** | **-** | **7.28** | **5.96** |  | **3.07** | **0.54** | **0.70** | **0.81** |

Average over 13 traits in 95 modern specimens (ears, cobs and kernels) and 123 archeological cobs (a. Cob) and 151 archeological kernels (a.Kern): leng, length; U.Dia, upper diameter; Me.Dia, medium diameter; Ma.Dia, maximum diameter; Inf.Dia, inferior diameter; N Row, number of row in ears and cobs; ker/Row, number of kernel per row in ears and cobs; t/kern, total number of kernels; wid, width of kernel; Thic, thickness of kernel; Per, perimeter, Are, area; SF, Shape factor; DF, Feret diameter. All measures are expressed in mm.
