# Supplementary material for: Genetic and phenotypic diversity in 2000 years old maize (Zea mays L.) samples from the Tarapacá region, Atacama Desert, Chile
Source: PLoS One. 2019 Jan 30;14(1):e0210369. doi: 10.1371/journal.pone.0210369 (PMC6353141; doi:10.1371/journal.pone.0210369)
Supplement: S2 Table — (DOCX) [file pone.0210369.s002.docx]

**S2 Table. Microsatellites used in this study in archaeological and modern kernel samples.**

| **Locus** | **Chro. Bin** | **5'- 3'** | **Type** | | **RM** | | **A.Tem (°C)** | |
| --- | --- | --- | --- | --- | --- | --- | --- | --- |
| phi056 | 1 | [ACTTGCTTGCCTGCCGTTAC](http://www.maizegdb.org/data_center/primer/111679)  [CGCACACCACTTCCCAGAA](http://www.maizegdb.org/data_center/primer/111680) | Trinucleotide | CCG | | 55 | |  |
| phi029 | 3.04 | TTGTCTTTCTTCCTCCACAAGCAGCGAA  TTTCCAGTTGCCACCGACGAAGAACTT | Complex | AG/AGAC | | 56 | |  |
| umc1332 | [5.04](http://www.maizegdb.org/bin_viewer?bin=5&sub=4) | [CCTCTTGCTTCCTCGTCATGTACT](http://www.maizegdb.org/data_center/primer/248595) [AAGGAGCTGGAACATAAAACACCA](http://www.maizegdb.org/data_center/primer/248596) | Trinucleotide | CTA | | 61 | |  |
| phi075 | 6.0 | GGAGGAGCTCACCGGCGCATAA  AAAGGTTACTGGACAAATATGCGTAACTCA | Dinucleotide | CT | | 55 | |  |
| phi034 | [7.02](http://www.maizegdb.org/bin_viewer?bin=7&sub=2) | [TAGCGACAGGATGGCCTCTTCT](http://www.maizegdb.org/data_center/primer/111671)  [GGGGAGCACGCCTTCGTTCT](http://www.maizegdb.org/data_center/primer/111672) | Trinucleotide | CTT | | 61 | |  |
| phi059 | [10.2](http://www.maizegdb.org/bin_viewer?bin=10&sub=2) | AAGCTAATTAAGGCCGGTCATCCC  TCCGTGTACTCGGCGGACTC | Trinucleotide | CCG | | 61 | |  |
| Phi127 | 2.08 | ATATGCATTGCCTGGAACTGGAAGGA  AATTCAAACACGCCTCCCGAGTGT | Tetranucleotide | AGAC | | 47 | |  |
| Phi063 | 10.02 | GGCGGCGGTGCTGGTAG  CAGCTAGCCGCTAGATATACGCT | Tetranucleotide | TACT | | 61 | |  |

Microsatellite markers analyzed in this study. Loc, Names of loci according to MaizeGDB; Chro.bin, Chromosome bin of SSR indicated; 5’-3’, Primers; Type, Number of repetitions of base pairs; RM, Repeat motif of SSR; A.Temp (°C), Annealing temperature in degrees Celsius for each SSR.
