# Supplementary material for: Genetic and phenotypic diversity in 2000 years old maize (Zea mays L.) samples from the Tarapacá region, Atacama Desert, Chile
Source: PLoS One. 2019 Jan 30;14(1):e0210369. doi: 10.1371/journal.pone.0210369 (PMC6353141; doi:10.1371/journal.pone.0210369)
Supplement: S3 Table — (DOCX) [file pone.0210369.s003.docx]

**S3. Table. aDNA readings and concentrations measured in NANODROP and QUBIT.**

| **ID** | **280/260** | **260/230** | **ND**  **ng/µl** | **QT**  **ng/µl** |
| --- | --- | --- | --- | --- |
| *TV260 | 1.95 | 1.52 | 1388.3 | 89.2 |
| 229B | 1.94 | 1.87 | 1935.2 | 76.4 |
| *T13218 | 1.89 | 2.1 | 3675.1 | 67.8 |
| *TV195 | 1.98 | 1.09 | 1035.7 | 31.6 |
| 222 | 1.89 | 3.6 | 247.8 | ^+^nr |
| *T13229A | 1.76 | 1.61 | 1292.2 | 89 |
| *TV200 | 1.8 | 2.11 | 4257.9 | 96.6 |
| *T13223 | 1.88 | 1.63 | 1705.7 | 73 |
| *T13226 | 1.93 | 1.89 | 2157.2 | 79.4 |
| *PT6 | 1.72 | 1.13 | 820.8 | 96.4 |
| *TV184 | 1.9 | 1.37 | 508.3 | 60.8 |
| 150 | 1.28 | 0.58 | 53.5 | ^+^nr |
| PT5 | 1.52 | 1.32 | 336 | ^+^nr |
| PT4 | 1.5 | 1.57 | 355 | ^+^nr |
| PT1 | 1.82 | 1.43 | 513.1 | ^+^nr |
| 216 | 1.43 | 0.77 | 784 | ^+^nr |
| *PT3 | 1.77 | 1.69 | 886.5 | 113 |
| *TV137 | 1.79 | 1.69 | 53.5 | 20.2 |
| 150 | 1.28 | 0.58 | 53.5 | ^+^nr |
| 222 | 1.3 | 0.68 | 54.4 | ^+^nr |
| PT2 | 1.31 | 0.72 | 152.8 | ^+^nr |
| 236 | 1.53 | 1.54 | 503 | ^+^nr |
| *TV259 | 1.98 | 2.15 | 2580.74 | 108 |
| **HTr13-1 | 0.98 | 0.40 | 73.06 | 5.0 |
| **HTr13-2 | ^+^nr | ^+^nr | ^+^nr | ^+^nr |
| **HT13-3 | ^+^nr | ^+^nr | ^+^nr | ^+^nr |
| **HT13-4 | ^+^nr | ^+^nr | ^+^nr | ^+^nr |
| **HT13-5 | ^+^nr | ^+^nr | ^+^nr | ^+^nr |
| **HT13-6 | ^+^nr | ^+^nr | ^+^nr | ^+^nr |

DNA concentrations of archaeological sample measured on NANODROP and QUBIT for 21 archaeological kerns and six fragments of husk. Id, Identification number of archaeological sample; * kern samples used in the analysis;** fragment of husk used as a negative control to discard contamination; 260-280 Ratio of absorbance 260nm - 280nm; 230-260, Ratio of absorbance 230nm - 260nm; ND ng/ µl,DNA concentrations of NANODROP; QT ng/ µl, DNA concentrations on QUBIT.^+^no reading available.
