# Supplementary material for: Genetic and phenotypic diversity in 2000 years old maize (Zea mays L.) samples from the Tarapacá region, Atacama Desert, Chile
Source: PLoS One. 2019 Jan 30;14(1):e0210369. doi: 10.1371/journal.pone.0210369 (PMC6353141; doi:10.1371/journal.pone.0210369)
Supplement: S4 Table — (DOCX) [file pone.0210369.s004.docx]

**S4. DNA Amplification Protocol**

Amplification of DNA samples protocol was carried out by Polymerase Chain Reaction (PCR) under the following conditions: 0.1 µl Go Taq (Go Taq® Flexi DNA Polymerase Promega), 2 µl green buffer, 2 µl MgCl, 0.5 µl BSA, 0.1 µl forward primer, 0.2 µl reverse primer, 1 µl each dNTP, 0.1 µl fluorescent dyes (VIC, NED, FAM, PET), 3 µl ADN (60 µl/ng) and 0.9 µl nuclease free water (Promega). PCR was performed under the following conditions: an initial denaturation cycle at 95 °C for 3 minutes, followed by a 30 denaturation cycles at 94 °C for 30 seconds, one annealing cycle with temperatures between 47 °C and 61 °C (Fig. S2) for 30 seconds, a cycle extension at 72 °C for 1.5 minutes, a final elongation at 72 °C for 10 minutes and a hold cycle to 10 °C ∞. The PCR products were checked in agarose gel (1.5% ammonium bromide) in 1X TAE buffer (0.04M Tris acetate, 0.001 M EDTA, pH 8.0) under electrophoresis and visualized under
