# Supplementary material for: Genetic and phenotypic diversity in 2000 years old maize (Zea mays L.) samples from the Tarapacá region, Atacama Desert, Chile
Source: PLoS One. 2019 Jan 30;14(1):e0210369. doi: 10.1371/journal.pone.0210369 (PMC6353141; doi:10.1371/journal.pone.0210369)
Supplement: S5 Table — (DOCX) [file pone.0210369.s005.docx]

**S5 Table DNA size range amplification in modern and archaeological samples**

| **SSR** | **Ho** | **He** | **DNA size (bp)** | **N allele** | **aDNA size (bp)** |
| --- | --- | --- | --- | --- | --- |
| **Phi127** | 0.168 | 0.409 | 120-124-128-132-140 | 5 | 128 |
| **Phi063** | 0.117 | 0.579 | 168-172-176-180-184-188-192-196-228 | 9 | 192 |
| **Phi059** | 0.409 | 0.462 | 167-170-176-179 | 4 | 167-176 |
| **Phi029** | 0.568 | 0.553 | 156-162-166-171-174 | 5 | *158*-166-171 |
| **Umc1332** | 0.578 | 0.564 | 141-162-165-168 | 4 | 141-*153*-162-165 |
| **Phi075** | 0.271 | 0.391 | 248-250-260-262-270 | 5 | *258*-262-*288* |
| **Phi034** | 0.606 | 0.532 | 144-147-150-153-159 | 4 | 159 |
| **Phi056** | 0.586 | 0.681 | 259-262-265-268-271 | 5 | 259-262-268 |
| **Average** | 0.413 | 0.521 |  | 5.1 |  |

SSR, Simple Sequence Repeats or microsatellites used in this study; Ho, observed heterozygosity for six modern populations; He, expected heterozygosity in six modern population; DNA size (bp), size range amplification of 95 specimens in modern samples; N alleles, number of alleles per locus in modern samples; aDNA size (bp), size range amplification in archeological samples from 12 specimens.
