# Supplementary material for: Genetic and phenotypic diversity in 2000 years old maize (Zea mays L.) samples from the Tarapacá region, Atacama Desert, Chile
Source: PLoS One. 2019 Jan 30;14(1):e0210369. doi: 10.1371/journal.pone.0210369 (PMC6353141; doi:10.1371/journal.pone.0210369)
Supplement: S6 Table — (DOCX) [file pone.0210369.s006.docx]

**S6 Table DNa size range comparison with other modern and archaeological Andean maize.**

| **SSR** | **Camus-Kundailevi et al. 2006**  **(mDNA)** | **Lia et al. 2007 (aDNA )** | **Lia et al. 2007 (mDNA)** | **Grimaldo 2011**  **(aDNA )** | **Grimaldo 2011**  **(mDNA )** | **aDNA**  **from Tarapacá** | **mDNA from Tarapacá** |
| --- | --- | --- | --- | --- | --- | --- | --- |
| Phi029 | 145-147-149-151-153-156-158-163-165-149-162 | 154 | 150-151-154-156-158-159-160-161 | 165-169-176 | 159-163-165-166-169-171-173-175-176 | *158*-166-171- | 156-162-166-171-174 |
| Phi034 | 119-122-133-136-138-139-142-145 | a |  | 137-147-155 | 140-150-153-155-156-158-159-162 | 159 | 144-147-150-153-159 |
| Phi059 | 115-145-150-152-155-159 | 157 | 148-152-154-157-161 | 162-171 | 161-165-167-170-174 | 167-176 | 167-170-176-179 |
| Phi056 | A | a |  | A | 250-253-256-259-262-265-268-271 | 259-262-268 | 259-262-265-268-271 |
| Phi063 | A | a |  | A | 172-177-179-180-184-188-192-196-208-228-240-248-256 | 192 | 168-172-176-180-184-188-192-196-228 |
| Phi075 | A | a |  | A | 227-241-243-245-246-247-250-254-256-258-260 | *258*-262-*288* | 248-250-260-262-270 |
| Phi127 | 111-113-119-124-126 | 112 | 112-114-120-124-126 | A | a | 128 | 120-124-128-132-140 |
| umc1332 | A | a |  | 139-160-163-181 | 133-136-154-157-160-163 | 141-*153*-162-165 | 141-162-165-168 |

Comparison between previous studies and Tarapacá samples. aDna=arcaheological Dna, mDna=modern Dna. Samples from Camus-Kulandailevi et al. 2006 are from America and Europe inbreed lines; Lia et al. 2007 archaeological and modern samples are from North Western of Argentina, Grimaldo archaeological samples are from north coast of Perú and NorthWestern Argentina and modern samples are primitive and historic landraces from Perú and Brazil.
